# In vivo connection imaging revealed distinct feedforward and intrinsic neurons in posterior inferotemporal cortex

**DOI:** 10.1186/1471-2202-14-S1-P296

**Published:** 2013-07-08

**Authors:** Noritaka Ichiohe, Elena Borra, Kathleen S Rockland

**Affiliations:** 1 Department of Ultrastructural Research, National Institute of Neuroscience, National Center of Neurology and Psychiatry, Kodaira, 187-8502, Tokyo, Japan; 2 Laboratory for Cortical Organization and Systematics, RIKEN Brain Science Institute, Wako City, Saitama, 351-0198, Japan

## 

We investigated [1] circuits for object recognition in macaque anterior (TE) and posterior inferotemporal cortex (TEO), using a two-step method for in vivo anatomical imaging. In step 1, red fluorescent tracer was injected into TE to reveal and Pre-target patches of feedforward neurons in TEO. In step 2, these were visualized on the cortical surface in vivo, and injected with green fluorescent tracer. Histological processing revealed that patches >500 μm from the injection site in TEO consisted of intermingled green TEO-TE intrinsically projecting neurons and red TEO-to-TE neurons, with only few double-labeled neurons. In contrast, patches near the injection site in TEO contained many double-labeled neurons. Two parallel, spatially intermingled circuits are suggested: (1) TEO neurons having very local intrinsic collaterals and projection to TE (2) TEO neurons projecting more widely in the intrinsic network, but not to TE. These parallel systems might be specialized for, respectively, fast vs. highly processed signals.
